# Supplementary material for: Micro RNA Transcriptome Profile in Canine Oral Melanoma
Source: Int J Mol Sci. 2019 Sep 28;20(19):4832. doi: 10.3390/ijms20194832 (PMC6801976; doi:10.3390/ijms20194832)
Supplement: Supplementary file 1 [file ijms-20-04832-s001.zip › Supplementary figures/Figure S1-3.pdf]

MiRNA profile in canine oral melanoma

Md. Mahfuzur Rahman, Yu-Chang Lai, Norio Ushio, Al Asmaul Husna, Hui-wen Chen, Yukiko Tanaka, Noriaki Miyoshi, Takayuki Nakagawa, Ryuji Fukushima, Naoki Miura

(a)

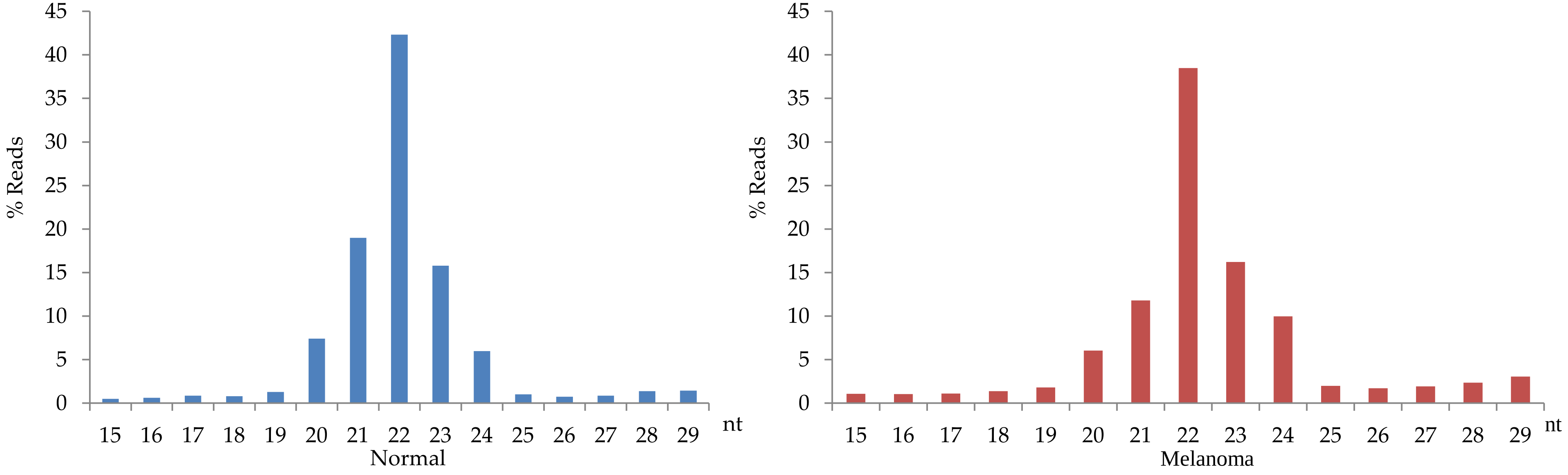

(b)

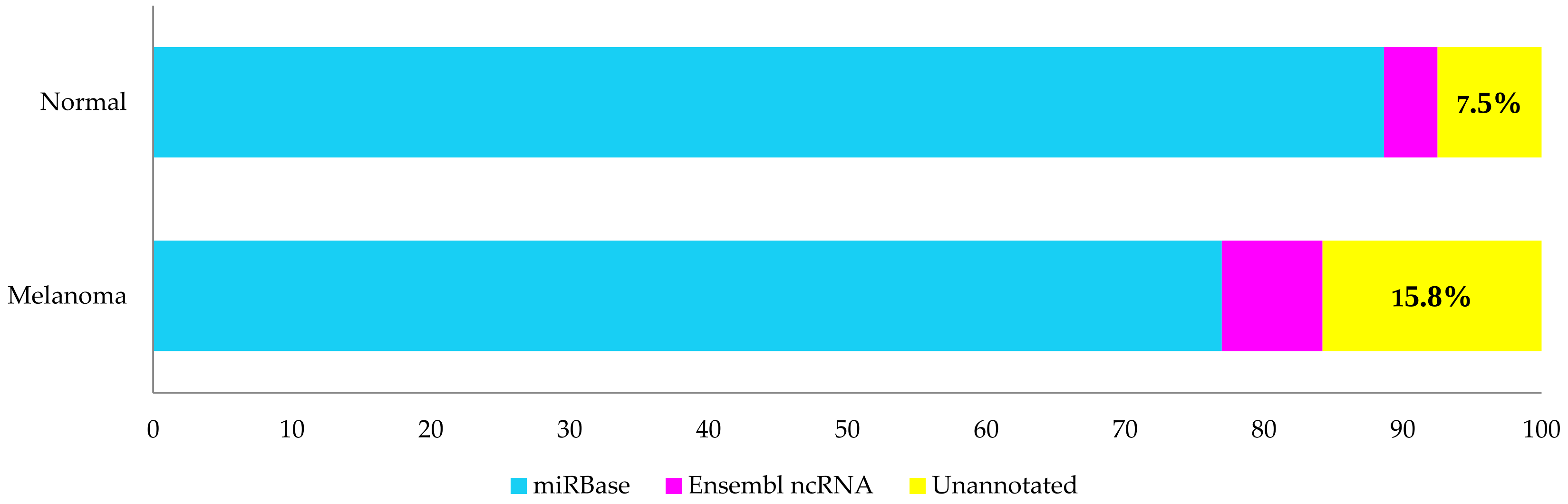

Normal
Melanoma
7.5%
15.8%
0
10
20
30
40
50
60
70
80
90
100
miRBase
Ensembl ncRNA
Unannotated

(c)

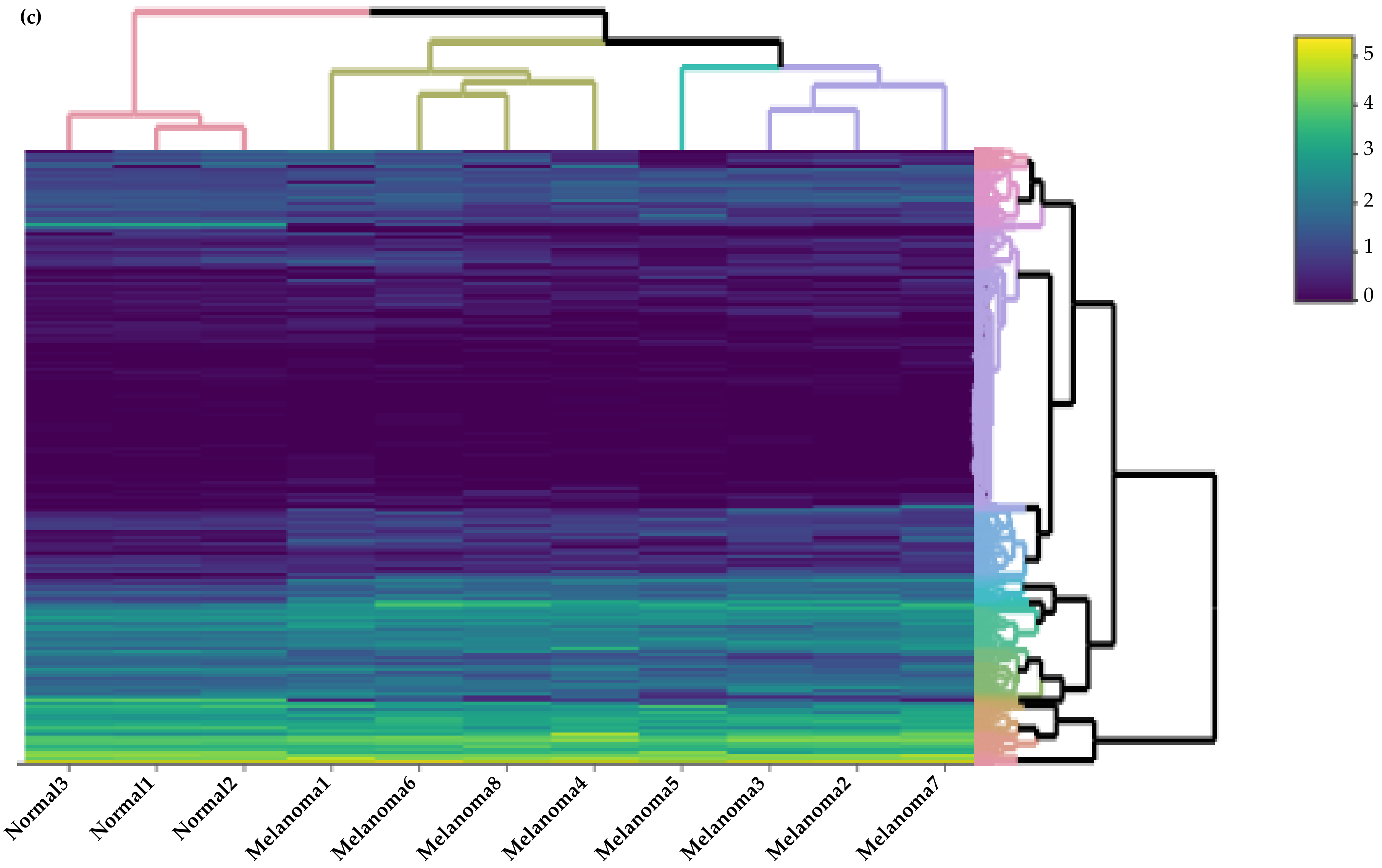
5
4
3
2
1
0
Normal3
Normal1
Normal2
Melanoma1
Melanoma6
Melanoma8
Melanoma4
Melanoma5
Melanoma3
Melanoma2
Melanoma7

(d)

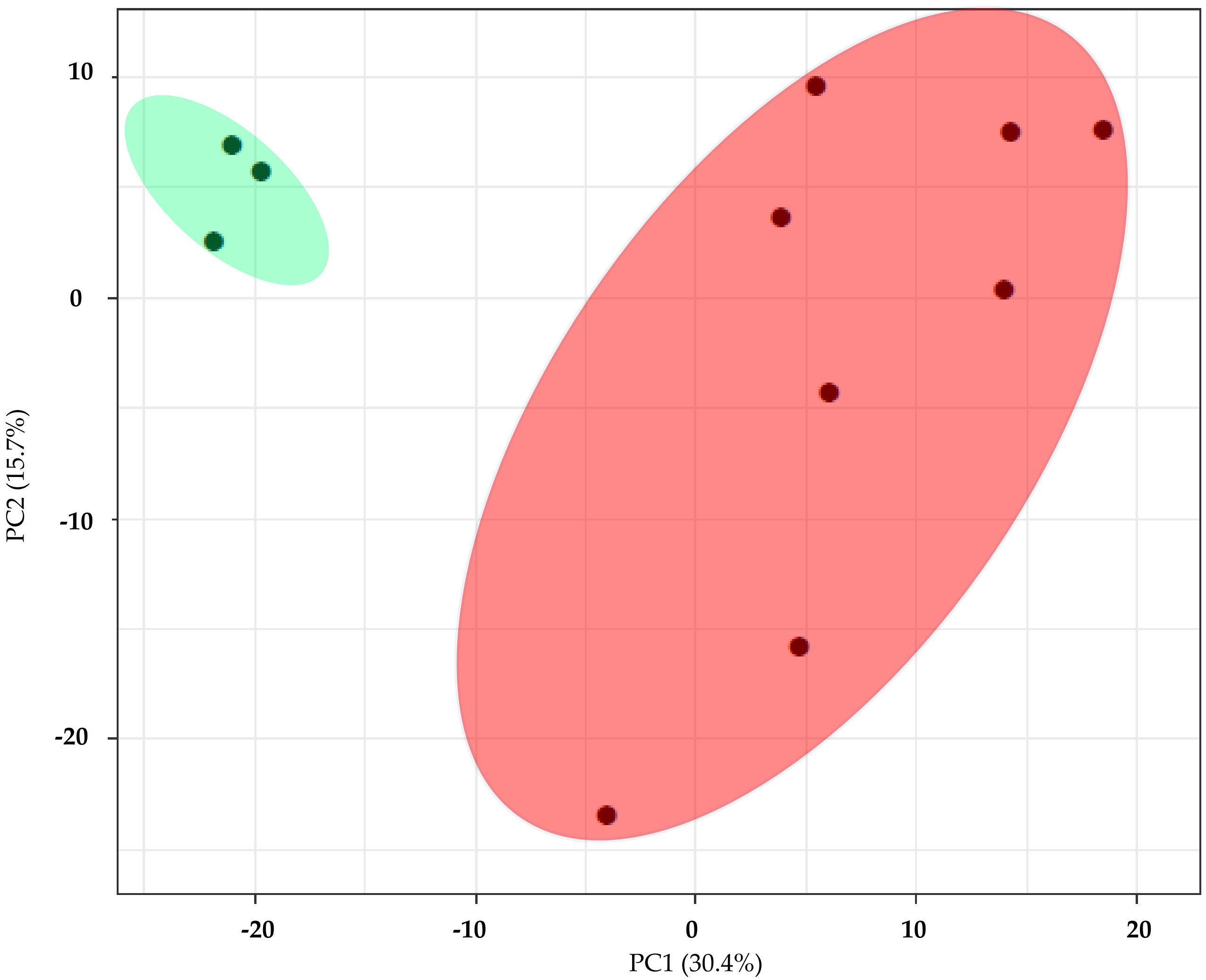

Figure S1. Profile of small RNA reads in canine oral melanoma: **(a)** Length distribution of clean reads in the normal and melanoma libraries; **(b)** Percentages of the clean reads annotated by the miRBase and Ensembl non-coding RNA databases; c) Unsupervised Euclidean hierarchical clustering by the miRNA normalized expression values in the normal and melanoma libraries, The colour scale (upper right) indicates the expression values; **(d)** Principal component analysis (PCA) of normal (green) and melanoma (pink) samples. The miRNAs read counts were normalized and transformed before PCA.

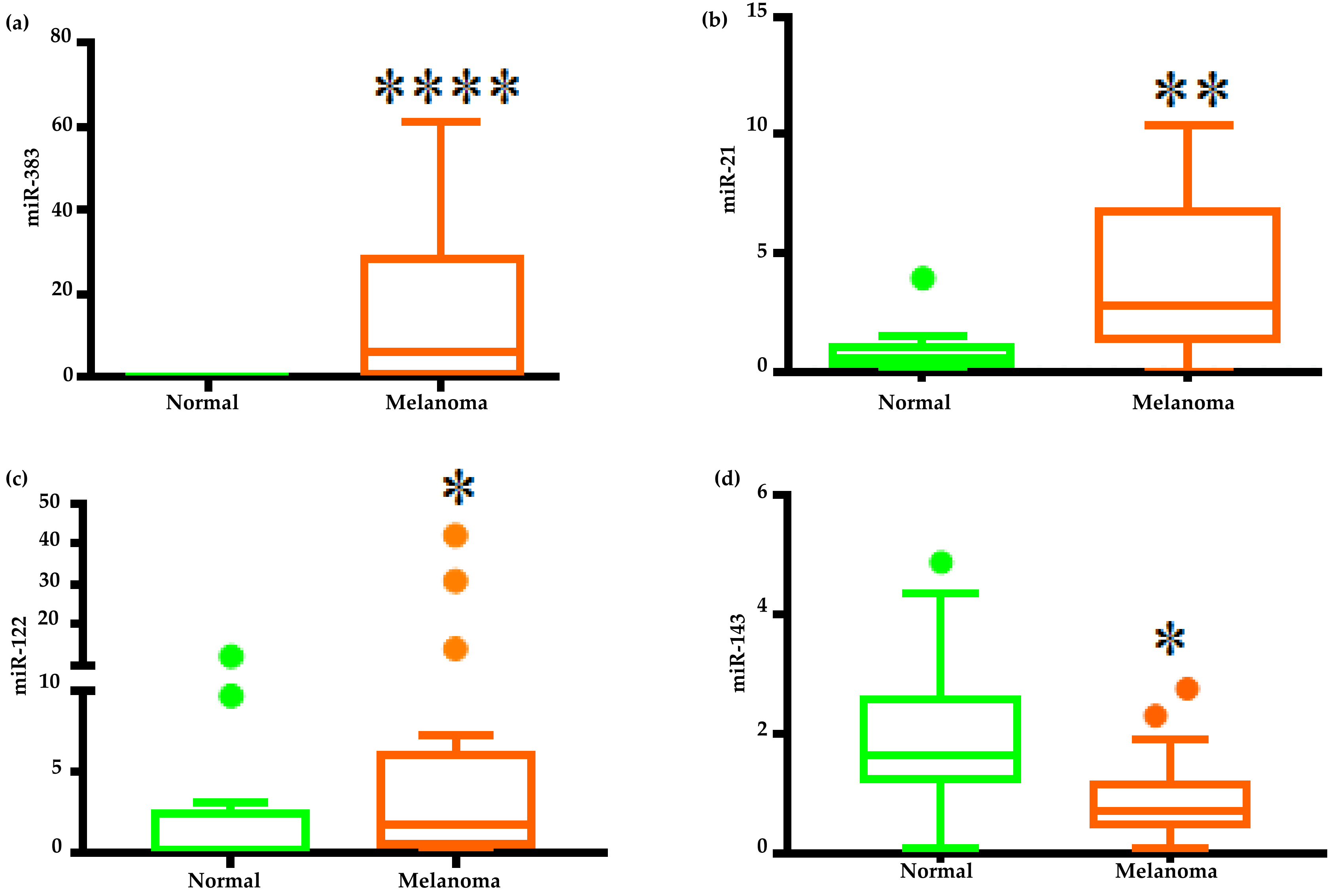

Figure S2. Differential miRNA expression in COM. (a-c) Relative expression of up-regulated miR-383, 21 and 122; (d) Relative expression of down-regulated miR-143. The Y-axes indicates the relative miRNA expression levels normalized against RNU6B (normal n=12, melanoma n=17; Mann-Whitney test followed by Tukey's test; *P <0.05, **P <0.01, ****P <0.0001)

(a)

Dog PAX9 ENST00000361487.6   3′ UTR length: 4013

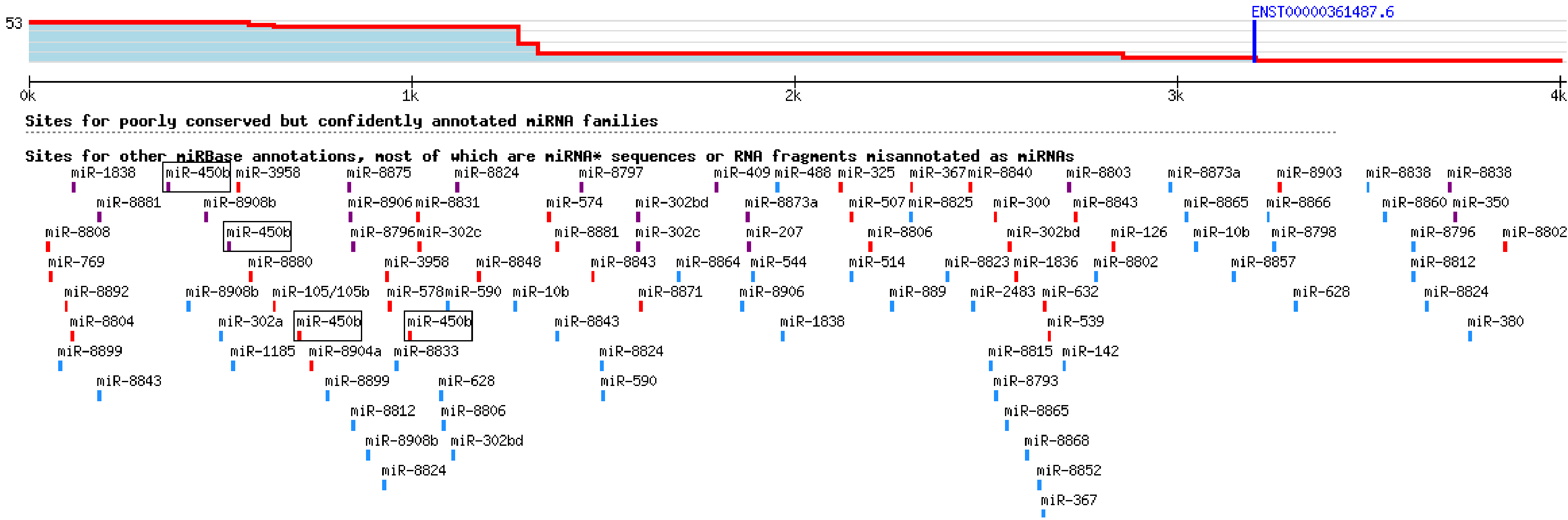

	Predicted consequential pairing of target region (top) and miRNA (bottom)	Site type	Context++ score	Context++ score percentile	Weighted context++ score	Conserved branch length	P_{CT}
Position 380-387 of PAX9 3' UTR<br>cfa-miR-450b	5' ...GCAAUUGUUGAGAUUUUGCAAAA...<br>||||||| 3' UAAGUCCUUGUAUAACGUUUU	8mer	-0.22	96	-0.22	0.153	N/A
Position 544-551 of PAX9 3' UTR<br>cfa-miR-450b	5' ...AGAUGACCUAUUUUGUUGCAAAA...<br>||||||| 3' UAAGUCCUUGUAUAACGUUUU	8mer	-0.19	94	-0.19	0	N/A
Position 735-741 of PAX9 3' UTR<br>cfa-miR-450b	5' ...UUUUUUUCUUUCUUUUUGCAAAG...<br>||||||| 3' UAAGUCCUUGUAUAACGUUUU	7mer-m8	-0.03	50	-0.03	0.233	N/A
Position 1033-1039 of PAX9 3' UTR<br>cfa-miR-450b	5' ...UUAAUGUGAAACGCUUUGCAAAG...<br>||| ||||||| 3' UAAGUCCUUGUAU-AACGUUUU	7mer-m8	-0.08	78	-0.07	0.153	N/A

```
       .......350.......360...........370...........380............390.......400...............410.................420.......430.......440.....
Dog    UCAACCUGAACUUUUGAAACGUGCAA---UUGUU---GAGA-UUUUGC-AAAAUC---AAUAAAGGAAACUACAU------AUAGAAAA------AAAA----AGUUAUGCUAUACCCUCUAAUCAAAUAA-
Human  UCAACCUGAACUUUUGAAAUGUGCAA---UUGUU---GAGA-UUUUGC-AAAAUC---AAUAAAGGAAAAUACUU------AUAG---------AAAA----AAUUAUGCUACACCCUCUAAUCAAAUAU-
```

(b)

Dog ACVR2A ENST00000241416.7 3′ UTR length: 3560

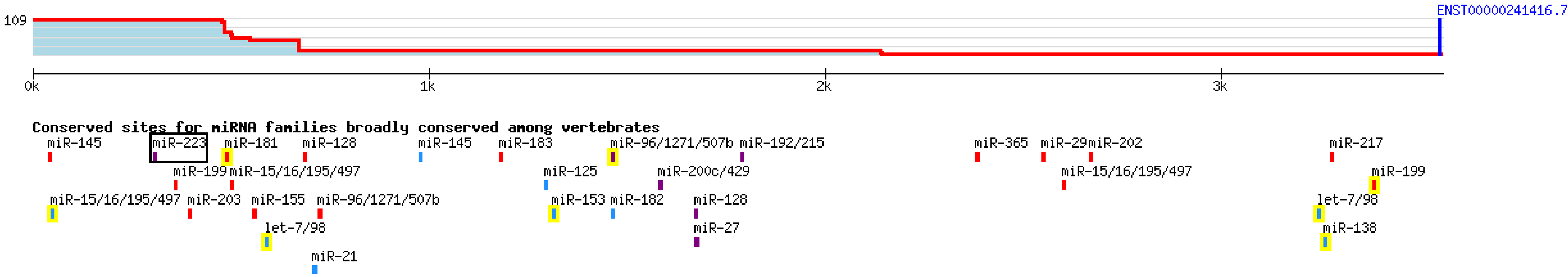

	Predicted consequential pairing of target region (top) and miRNA (bottom)	Site type	Context++ score	Context++ score percentile	Weighted context++ score	Conserved branch length	P_{CT}
Position 305-312 of ACVR2A 3' UTR<br>cfa-miR-223	5' ...AAUCAAGUGUUUGAA-AACUGACA... ||||| ||||||| 3' CCCCAUAAACUGUUUGACUGU	8mer	-0.46	98	-0.46	3.283	0.29

```
       260...........270........280..........290........300.........310.......320.............330.............340........350.......360........
Dog    ICUCCAAAU--C---AAGGAUC-UUUUGGACCU--GGCU-AAUCAAGUGU-UUG--AAAACUGACAUCAGAUUUC-----UAAAUGUCUGUCGG---AA---GACACUAAUUCC-UUAAAUGAACUACUGCUA---
Human  ICUCCAAAU--C---AAGGAUC-UUUUGGACCU--GGCU-AAUGGAGUGU-UUG--AAAACUGACAUCAGAUUUC-----UUAAUGUCUGUCAG---AA---GACACUAAUUCC-UUAAAUGAACUACUGCU----
```

(c)

Dog NDRG2 ENST00000298687.5 3′ UTR length: 852

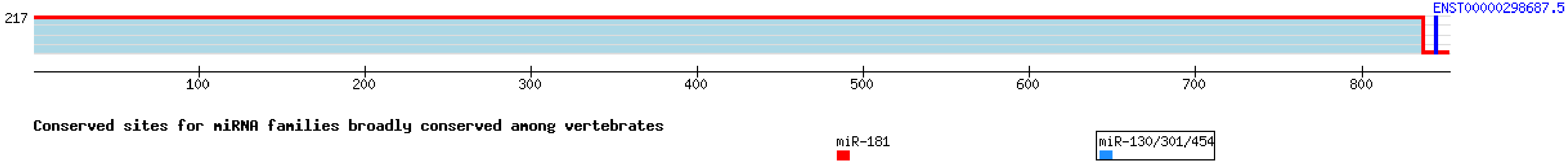

	Predicted consequential pairing of target region (top) and miRNA (bottom)	Site type	Context++ score	Context++ score percentile	Weighted context++ score	Conserved branch length	P_{CT}
Position 642-648 of NDRG2 3' UTR<br>cfa-miR-130a	5' ...CCUGGGGCCAUUGGCUGCACUAA...<br>|||||| 3' UACGGGAAAAUUGUAACGUGAC	7mer-A1	-0.16	80	-0.16	3.672	0.58
Position 642-648 of NDRG2 3' UTR<br>cfa-miR-301b	5' ...CCUGGGGCCAUUGGCUGCACUAA...<br>|||||| 3' CGAAACUGUUAUAGUAACGUGAC	7mer-A1	-0.16	80	-0.16	3.672	0.58
Position 642-648 of NDRG2 3' UTR<br>cfa-miR-301a	5' ...CCUGGGGCCAUUGGCUGCACUAA...<br>|||||| 3' CGAAACUGUUAUGAUAACGUGAC	7mer-A1	-0.16	80	-0.16	3.672	0.58

```
      .......................630.................640.......650............660.......670.......680..............690................700........
Dog   ----------------------UGG-GG--------CCAU-UGGCUGCACUAACU----UUGGUA-GCUGUGUAGACAGUGUGUGUCUA----CAGUGGGAGG-GG---G-A-GGGAG------c-----ucagc
Human ----------------------UGG-GG--------CCGUUUGGCUGCACUAACU----UUGGUA-GCUC-------AGUGUGCAUCUA----GAGUGGGACUGGG---G-A-GGGAG------c-----uaagc
```

Figure S3. Predicted conserved target binding site of miR-450b-PAX9, miR-223-ACVR2A and miR-301a-NDRG2 from TargetScan: **(a-c)**. Predicted binding sites of respective miRNA-mRNA are conserved between human and dog.
