# Supplementary material for: Micro RNA Transcriptome Profile in Canine Oral Melanoma
Source: Int J Mol Sci. 2019 Sep 28;20(19):4832. doi: 10.3390/ijms20194832 (PMC6801976; doi:10.3390/ijms20194832)
Supplement: Supplementary file 1 [file ijms-20-04832-s001.zip › Supplementary tables/Table S1a-b.docx]

**MiRNA profile in canine oral melanoma**

**Md. Mahfuzur Rahman, Yu-Chang Lai, Norio Ushio, Al Asmaul Husna, Hui-wen Chen, Yukiko Tanaka, Noriaki Miyoshi, Takayuki Nakagawa, Ryuji Fukushima, Naoki Miura**

**Table S1a**. Signalment characteristics and WHO clinical stage of the 17 canine oral malignant melanomas

| **COM tissue specimen for NGS and qPCR** | | | | | |
| --- | --- | --- | --- | --- | --- |
| No | Age (Years) | Sex | Breed | WHO Stage | NGS(N) or qPCR (q) |
| T1 | 12.7 | Male | Miniature | Ⅳ | N and q |
| T2 | 14.8 | Male | Mongrel | Ⅳ | N and q |
| T3 | 10 | Male | Golden  Retriever | Ⅳ | N and q |
| T4 | 10.11 | Male | Miniature | Ⅰ | q |
| T5 | 7.11 | Male | Miniature | Ⅰ | q |
| T6 | 10.9 | Male | Miniature | Ⅳ | q |
| T7 | 12 | Male | Shiba | Ⅳ | q |
| T8 | 13 | Male | Pomerania | Ⅰ | q |
| T9 | 10.3 | Male | Yorkshire | Ⅳ | q |
| T10 | 10.2 | Male | Chiwawa | Ⅳ | N and q |
| T11 | 12.4 | Female | Miniature | Ⅳ | q |
| T12 | 14.6 | Female | Miniature | Ⅱ | q |
| T13 | 15.2 | Female | Mongrel | Ⅳ | N and q |
| T14 | 12.11 | Male | Miniature | Ⅳ | N and q |
| T15 | 12.4 | Male | Shiba and  Miniature cross | Ⅳ | N and q |
| T16 | 15.2 | Female | Mongrel | Ⅳ | N and q |
| T17 | 10.8 | Male | Miniature | Ⅳ | q |

**Table S1b.** Clean sequencing reads from the normal and melanoma individual libraries

| **Column ID** | **Normal** | | |
| --- | --- | --- | --- |
| Sample Name | Reads |  | Reads After adapter treaming |
| Nomal1 | 31719088 |  | 19015734 |
| Nomal2 | 34437764 |  | 15742516 |
| Nomal3 | 37308370 |  | 16280411 |
| Total | 103465222 |  | 51038661 |
|  | Melanoma | | |
| Melanoma1 | 29554264 |  | 15993157 |
| Melanoma2 | 28585813 |  | 13852584 |
| Melanoma3 | 28264207 |  | 10101754 |
| Melanoma4 | 27946191 |  | 11432175 |
| Melanoma5 | 29796066 |  | 13157883 |
| Melanoma6 | 36882368 |  | 22866409 |
| Melanoma7 | 57273744 |  | 42202229 |
| Melanoma8 | 27785482 |  | 12462538 |
| Total reads | 266088135 |  | 142068729 |
