# Supplementary material for: Micro RNA Transcriptome Profile in Canine Oral Melanoma
Source: Int J Mol Sci. 2019 Sep 28;20(19):4832. doi: 10.3390/ijms20194832 (PMC6801976; doi:10.3390/ijms20194832)
Supplement: Supplementary file 1 [file ijms-20-04832-s001.zip › Supplementary tables/Table S2.docx]

**Table S2.** Differentially expressed microRNAs in canine oral melanoma

| **miR** | **Stand** | **Species** | **FC** ^1^ | **FDR** ^2^ | **Chr** ^3^ |
| --- | --- | --- | --- | --- | --- |
| mir-8884 | Mature 3' | Canis familiaris | 4.03 | 3.46E-02 | 8 |
| mir-107 | Mature 3' | Canis familiaris | 4.25 | 2.1E-02 | 28 |
| mir-8859b | Mature 3' | Canis familiaris | 4.28 | 2.79E-02 | 18 |
| mir-421 | Mature 3' | Canis familiaris | 4.41 | 2.78E-02 | X |
| mir-190a | Mature 5' | Canis familiaris | 5.33 | 4.45E-02 | 30 |
| mir-454 | Mature 3' | Homo sapiens | 5.69 | 1.27E-02 | 9 |
| mir-335 | Mature 5' | Canis familiaris | 5.77 | 1.77E-02 | 14 |
| mir-132 | Mature 3' | Homo sapiens | 5.97 | 5.93E-03 | 9 |
| mir-301a | Mature 3' | Canis familiaris | 6.88 | 2.5E-03 | 9 |
| mir-330 | Mature 5' | Canis familiaris | 7.19 | 2.06E-03 | 1 |
| mir-21 | Mature 5' | Homosapiens//Canis familiaris | 7.60 | 4.2E-04 | 9 |
| mir-423//mir-423a | Mature 5' | Homosapiens//Canis familiaris | 8.69 | 5.81E-03 | 9 |
| mir-363 | Mature 3' | Canis familiaris | 9.62 | 3.00E-04 | X |
| mir-146b | Mature 5' | Homosapiens//Canis familiaris | 15.32 | 1.62E-03 | 28 |
| mir-140 | Mature 3' | Homo sapiens | 23.03 | 6.27E-03 | 5 |
| mir-9-2//mir-9-3//mir-9-1 | Mature 5' | Canis familiaris | 62.71 | 3.36E-07 | 3,7 |
| mir-223 | Mature 3' | Canis familiaris | 37.49 | 8.16E-04 | X |
| mir-383 | Mature 5' | Homosapiens//Canis familiaris | 207.48 | 2.43E-04 | 16 |
| mir-450b | Mature 5' | Canis familiaris | 60.14 | 3.09E-08 | X |
| mir-196a-2 | Mature 5' | Canis familiaris | 990.94 | 2.54E-08 | 27 |
| mir-371 | Mature 5' | Canis familiaris | 22.75 | 5.67E-05 | 1 |
| mir-542 | Mature 3' | Canis familiaris | 131.06 | 1.80E-09 | X |
| mir-130b | Mature 3' | Canis familiaris | 14.17 | 9.51E-05 | 26 |
| mir-424 | Mature 5' | Homo sapiens | 57.02 | 4.06E-09 | X |
| mir-450a | Mature 5' | Canis familiaris | 24.21 | 4.06E-05 | X |
| mir-106a | Mature 5' | Homo sapiens | 17.84 | 1.27E-05 | X |
| mir-301b | Mature 3' | Canis familiaris | 13.51 | 7.86E-05 | 26 |
| mir-20b | Mature 5' | Canis familiaris | 38.45 | 3.51E-07 | X |
| mir-18b | Mature 5' | Canis familiaris | 27.73 | 7.00E-06 | X |
| mir-424 | Mature 3' | Homosapiens//Canis familiaris | 71.56 | 8.27E-07 | X |
| let-7a-1//let-7a-3//let-7a-2 | Mature 5' | Homosapiens//Canis familiaris | -3.27 | 1.58E-02 | 10,5 |
| mir-126 | Mature 5' | Homosapiens//Canis familiaris | -3.56 | 1.75E-02 | 9 |
| mir-125a | Mature 5' | Canis familiaris | -4.56 | 6.52E-03 | 1 |
| let-7b | Mature 5' | Canis familiaris | -3.40 | 1.66E-02 | 10 |
| mir-101-2//mir-101-1 | Mature 3' | Canis familiaris | -3.19 | 1.71E-02 | 1,5 |
| mir-1271 | Mature 5' | Canis familiaris | -4.32 | 1.72E-02 | 4 |
| mir-183 | Mature 5' | Homosapiens//Canis familiaris | -3.55 | 2.25E-02 | 14 |
| mir-26b | Mature 5' | Homo sapiens | -2.76 | 4.42E-02 | 37 |
| mir-29c//mir-29c-1//mir-29c-2 | Mature 3' | Homosapiens//Canis familiaris | -4.20 | 3.68E-03 | 7 |
| mir-152 | Mature 3' | Homosapiens//Canis familiaris | -3.24 | 3.16E-02 | 9 |
| mir-1260a | Mature 5' | Homo sapiens | -3.43 | 2.72E-02 | 4 |
| mir-378i | Mature 5' | Homo sapiens | -3.11 | 3.27E-02 | 3 |
| mir-708 | Mature 5' | Homosapiens//Canis familiaris | -3.87 | 3.22E-02 | 21 |
| mir-31 | Mature 5' | Canis familiaris | -3.55 | 4.32E-02 | 11 |
| mir-143 | Mature 5' | Homo sapiens | -3.79 | 3.46E-02 | 4 |
| mir-1468 | Mature 5' | Canis familiaris | -5.32 | 2.69E-03 | X |
| mir-145 | Mature 3' | Homo sapiens | -5.54 | 6.97E-04 | 4 |
| let-7c | Mature 5' | Homosapiens//Canis familiaris | -5.91 | 2.88E-04 | 31 |
| mir-379 | Mature 5' | Homosapiens//Canis familiaris | -5.97 | 1.89E-02 | 8 |
| mir-147 | Mature 3' | Canis familiaris | -7.034 | 1.62E-03 | 30 |
| mir-96 | Mature 5' | Canis familiaris | -9.00 | 7.86E-05 | 14 |
| mir-99a-1//mir-99a-2 | Mature 5' | Canis familiaris | -12.07 | 4.9E-04 | 31,5 |
| mir-452 | Mature 5' | Canis familiaris | -16.75 | 9.86E-05 | X |
| mir-409 | Mature 5' | Homo sapiens | -16.85 | 2.12E-07 | 8 |
| mir-95 | Mature 3' | Canis familiaris | -17.61 | 1.12E-04 | 3 |
| mir-224 | Mature 5' | Canis familiaris | -28.17 | 1.14E-07 | X |
| mir-375 | Mature 3' | Canis familiaris | -38.64 | 6.83E-14 | 37 |
| mir-141 | Mature 3' | Homo sapiens | -153.25 | 0 | 27 |
| mir-429 | Mature 3' | Canis familiaris | -314.72 | 2.88E-22 | 5 |
| mir-205 | Mature 5' | Canis familiaris | -320.15 | 0 | 7 |
| mir-200c | Mature 3' | Homosapiens//Canis familiaris | -376.31 | 2.93E-22 | 27 |
| mir-200a | Mature 3' | Homo sapiens | -520.66 | 1.41E-27 | 5 |
| mir-200b | Mature 3' | Homo sapiens | -663.46 | 0 | 5 |
| mir-203a//mir-203 | Mature 3' | Homosapiens//Canis familiaris | -854.25 | 0 | 8 |

^1^ Fold change. ^2^ False discovery rate.  ^3^ Chromosome
