# Supplementary material for: Micro RNA Transcriptome Profile in Canine Oral Melanoma
Source: Int J Mol Sci. 2019 Sep 28;20(19):4832. doi: 10.3390/ijms20194832 (PMC6801976; doi:10.3390/ijms20194832)
Supplement: Supplementary file 1 [file ijms-20-04832-s001.zip › Supplementary tables/Table S4.docx]

**Table S4.** Over representation analysis of the down-regulated miRNAs target genes against cancer and other databases

| **Cancer Gene Census** | | |
| --- | --- | --- |
| Gene Set | Enrichments | P-Value |
| oncogene | 1.11 | 8.80E-2 |
| fusion | 1.04 | 4.42E-01 |
| others | 1.01 | 5.77E-01 |
| tumor suppresor genes | 0.84 | 9.66E-01 |
| **GLAD4U databases** | | |
|  |  |  |
| Cell Transformation, Neoplastic | 2.13 | 5.48E-12 |
| Neoplastic Processes | 1.73 | 5.64E-10 |
| Neoplasm Invasiveness | 1.81 | 5.95E-09 |
| Chromosome Aberrations | 1.66 | 3.59E-06 |
| Neoplasms, Squamous Cell | 1.53 | 7.39E-06 |
| Neoplasms | 1.40 | 8.42E-06 |
| Leukemia, Myeloid, Acute | 1.80 | 9.46E-06 |
| Neoplasm Metastasis | 1.55 | 1.12E-05 |
| Translocation, Genetic | 1.51 | 1.34E-05 |
| Leukemia | 1.52 | 1.58E-05 |
| **OMIM databases** | | |
| Colorectal cancer | 22.23 | 1.64E-06 |
| Diabetes mellitus, noninsulin-dependent | 12.88 | 4.82E-06 |
| Lung canceralveolar cell carcinoma, included | 18.31 | 4.87E-06 |
| Leukemia, acute myeloid | 14.82 | 1.52E-05 |
| Juvenile myelomonocytic leukemia | 37.35 | 3.87E-05 |
| Intervertebral disc disease | 31.12 | 7.64E-05 |
| Osteoporosis | 31.12 | 7.64E-05 |
| Tetralogy of fallot | 23.34 | 2.09E-04 |
| Breast cancer | 10.38 | 5.06E-04 |
| Myocardial infarction, susceptibility tomyocardial infarction | 15.56 | 7.85E-04 |
